# Evaluation of oral telmisartan administration as a suppression test for diagnosis of primary hyperaldosteronism in cats

**DOI:** 10.1111/jvim.16689

**Published:** 2023-03-29

**Authors:** Virginie Fabrès, Renaud Dumont, Mélanie Garcia, Dan Rosenberg, Benoit Rannou, Maxine Kurtz, Ghita Benchekroun

**Affiliations:** ^1^ Ecole Nationale Veterinaire d'Alfort, CHUVA Service de médecine interne Maisons‐Alfort Île‐de‐France F‐94700 France; ^2^ Centre Hospitalier Vétérinaire Aquivet Service de médecine interne F‐33320 Eysines (Bordeaux) France; ^3^ Centre Hospitalier Veterinaire Frégis Service de médecine interne 43 avenue Aristide Briand Arcueil 94110 France; ^4^ Micen Vet, Service de médecine interne Créteil France; ^5^ VetAgro Sup Service de Pathologie clinique, Marcy‐l'Etoile Auvergne‐Rhône‐Alpes France; ^6^ AzurVet‐Lab Laboratoire de biologie médicale St‐Laurent‐du‐Var France; ^7^ Ecole nationale vétérinaire d'Alfort, Univ Paris Est Créteil INSERM, IMRB Maisons‐Alfort F‐94700 France

**Keywords:** adrenal disorder, cardiology, cardiovascular, Conn disease, feline, hemodynamics, hypertension, hypokalemia

## Abstract

**Background:**

Development of a telmisartan‐based suppression test may facilitate the diagnosis of primary hyperaldosteronism (PHA) in cats, which remains difficult today.

**Objectives:**

To develop a telmisartan suppression test (TST) that is safe, and able to suppress aldosterone secretion in healthy cats but not in cats with PHA.

**Animals:**

Ten healthy cats and 6 cats with PHA.

**Methods:**

Prospective study using a placebo‐controlled crossover design to investigate a TST in healthy cats, and evaluation of TST in cats with PHA. Plasma aldosterone concentration, potassium concentration, and systolic blood pressure (SBP) were measured before (T0), and 1 hour (T1) and 1.5 hours after (T1.5) PO administration of 1 mg/kg of telmisartan, 2 mg/kg of telmisartan or placebo.

**Results:**

Median age in healthy cats was 3 years old (range, 1‐7). In healthy cats, a telmisartan dose of 2 mg/kg significantly decreased aldosterone concentration at T1 and T1.5 compared with T0. Placebo had no significant effect on aldosterone concentration. In cats diagnosed with PHA, a 2‐mg/kg dose of telmisartan did not induce any significant change in aldosterone concentration at T1 or T1.5 compared with T0. No adverse effects of telmisartan (e.g., hyperkalemia, systemic hypotension) were observed in any cats.

**Conclusions and Clinical Importance:**

The oral TST shows promise as a diagnostic test for the diagnosis of PHA in cats.

**Abbreviations**
ACE: angiotensin converting enzyme
ARB: angiotensin II receptor blocker
ARR: aldosterone‐to‐renin ratio
AVR: aldosterone variation rate
FST: fludrocortisone suppression test
PAC: plasma aldosterone concentration
PHA: primary hyperaldosteronism
PRA: plasma renin activity
RAAS: renin‐angiotensine‐aldosterone system
SBP: systolic blood pressure

## 1 INTRODUCTION

Primary hyperaldosteronism (PHA) is an adrenocortical disorder characterized by autonomous and excessive secretion of aldosterone as a result of unilateral or bilateral neoplasia, or bilateral hyperplasia of the zona glomerulosa of the adrenal gland.^1^ This mineralocorticoid excess can lead to hypokalemia or systemic hypertension or both and has been associated with progressive renal disease in cats.^2^ Cases are being reported more frequently, and understanding of the pathogenesis and clinical manifestations of the disease has improved.^1^, ^3^ However, diagnosis of PHA in cats remains challenging.^1^ The aldosterone‐to‐renin ratio (ARR) currently is considered the gold standard measurement for PHA screening,^1^ but assays for plasma renin activity (PRA) are not widely available, and stringent pre‐analytical conditions make its evaluation difficult. Moreover, evidence that PRA is suppressed in all cats with PHA is lacking. Currently, suspicion of PHA is based on a combination of suggestive clinical signs, an increased plasma aldosterone concentration (PAC) in the presence of otherwise unexplained hypokalemia, and visualization of an adrenal mass on ultrasound examination or computed tomography scan. However, in the case of bilateral adrenocortical hyperplasia, there may be minimal or no detectable change on ultrasonography, making PHA diagnosis even more challenging. For these reasons, a reliable and accessible confirmatory test is needed.

In human medicine, the 4 commonly used confirmatory tests for PHA are the fludrocortisone suppression test (FST), the saline infusion test, the captopril challenge test and the oral sodium loading test.^4^ The FST has already been evaluated in cats and a lack of aldosterone suppression was observed in 6/9 cats with PHA.^5^, ^6^ However, there are concerns that fludrocortisone could be detrimental to cats with PHA that have severe hypokalemia or severe hypertension or both. In elderly humans, who are considered to be a vulnerable population, an alternative suppression test that uses the selective angiotensin 2 type 1 receptor (AT1R) antagonist losartan has been demonstrated to be safe and accurate to confirm PHA.^7^ This test is quick, with only 1 blood sample required 2 hours after PO administration of losartan. Telmisartan is the only AT1R antagonist authorized for use in cats. It has proven to be an effective and safe antiproteinuric and antihypertensive drug at the dosage of 1 to 2 mg/kg PO q24h, respectively.^7^, ^8^ Given that angiotensin II (and hyperkalemia) are the most powerful stimuli for adrenocortical aldosterone secretion, we hypothesized that AT1R blockade would lead to a significant reduction in PAC in healthy cats but not in cats with PHA that have autonomous secretion of aldosterone.

Our aim was to evaluate the ability of PO telmisartan to suppress PAC in healthy cats, and to identify the dosage and time corresponding to optimal aldosterone suppression. The potential of a telmisartan suppression test to discriminate cats with PHA from healthy cats also was investigated.

## 2 MATERIALS AND METHODS

### 2.1 Cats

We enrolled 10 healthy student‐owned cats. Eligibility criteria were age ≥6 months, an unremarkable history and physical examination, plasma concentrations of creatinine, sodium, and potassium within reference intervals, urine specific gravity >1.030, and systolic blood pressure (SBP) ≤160 mm Hg.

Six client‐owned cats admitted to Veterinary Teaching Hospital of the National Veterinary School of Alfort and diagnosed with PHA also were enrolled between September 2016 and September 2018. The diagnosis of PHA was based on increased PAC (>540 pmol/L) associated with hypokalemia in the presence of an adrenal mass or increased ARR (>3.8 × 10^−9^).^8^ Exclusion criteria were concurrent treatment expected to affect the renined angiotensin aldosterone system (e.g., angiotensin converting enzyme inhibitor, angiotensin receptor blocker, aldosterone receptor antagonist, calcium channel blocker, diuretic) within the week before enrollment. In PHA cats that were hypertensive, the telmisartan suppression test was performed as soon as PHA was suspected and adequate medications were initiated after the test was completed.

### 2.2 Study design

The initial study in healthy cats was a randomized, placebo‐controlled, crossover study. At study baseline, all cats had a physical examination and SBP determination, plasma creatinine, sodium and potassium concentrations were measured, and urinalysis was performed.

For the telmisartan suppression test, healthy cats were given telmisartan solution PO (Semintra, 4 mg/mL, Boehringer Ingelheim) at a dose of 1 mg/kg, 2 mg/kg or the equivalent volume of the 2 mg/kg dose of tap water as a placebo. Blood samples were taken before (T0), and 1 hour (T1) and 1.5 hours (T1.5) after telmisartan or placebo administration for the measurement of plasma aldosterone and potassium concentrations. Systolic blood pressure (SBP) was measured at the same time, before blood sampling, to evaluate the safety of the test. The telmisartan dosage was chosen based on previous studies evaluating the antiproteinuric and antihypertensive efficacy and safety of telmisartan.^9^, ^10^ A 1‐week washout period was applied between each test.

Based on results in healthy cats, an uncontrolled follow‐on study of the best‐performing protocol for the suppression test was conducted on cats with PHA using only telmisartan at a dosage of 2 mg/kg.

The study protocol was approved by the scientific ethics committee of the National Veterinary School of Alfort (Comerc 2017‐09‐06) and all owners consented to their cats' participation in the study.

### 2.3 Systolic blood pressure measurement

Systolic blood pressure was measured in the hospital environment by the same trained observers (VF, RD) using the standard Doppler ultrasonography method (811‐BL, Parks Medical Electronics, Inc, Aloha, Oregon) according to American College of Veterinary Internal Medicine (ACVIM) guidelines.^11^ Cats were restrained as little as possible in sternal recumbency or in a sitting position. In healthy cats, measurement was always performed in the morning and in the same room to ensure minimal variability between measurements. Several measurements were taken over 5 to 10 minutes to obtain the average of 5 concordant values.

### 2.4 Sample collection and analysis

Blood samples were collected by jugular venipuncture into heparin‐coated tubes for the measurement of plasma sodium, potassium, and aldosterone concentrations. Sodium and potassium concentrations were measured using an electrode‐based analyzer (Smart 30 Vet, I‐Sens, France). Blood also was collected into ice‐chilled EDTA‐coated tubes and then centrifuged at 4°C for 8 minutes at 3000*g*. Plasma was stored at −20°C and −80°C pending measurement of aldosterone and PRA, respectively. Aldosterone concentration was determined using a validated radio‐immunoassay (RIAZENco Aldosterone, ZenTech) at the laboratory of the veterinary school of Lyon (Vetagrosup, Lyon, France). The aldosterone variation rate (AVR) was calculated at T1 and T1.5 using the formula: AVR = ([aldosterone]_T1 or T1.5_ − [aldosterone]_T0_)/[aldosterone]_T0_. The lowest value of AVR (i.e., the highest aldosterone inhibition) that occurred at either T1 or T1.5 then was determined. Plasma renin activity was measured at the Department of Nephrology of the University Medical Center, Utrecht, the Netherlands as previously described.^8^ In brief, plasma was incubated at pH 6.0 for 1 hour at 37°C in the presence of inhibitors of angiotensinases and angiotensin I converting enzyme. Samples then were deproteinized with acetone and ammonia and centrifuged. The supernatants were evaporated and redissolved in assay buffer, and angiotensin I was measured by radioimmunoassay. Plasma ARR was calculated as the ratio of PAC (pmol/L) to PRA (fmol/L/s).

### 2.5 Statistical analysis

Descriptive statistics were generated using Microsoft 365 Excel Data Analysis, and other statistical analyses were performed using Statistical Analysis System (SAS University Edition 9.04.01M6P11072018). Continuous variables are presented as median and range (minimum; maximum). The PAC, potassium concentration, and SBP at the different time points were compared using Wilcoxon's signed rank test. Comparisons of baseline potassium concentration, SBP, aldosterone concentration, and AVR between groups of healthy cats receiving placebo, healthy cats receiving telmisartan at 1 and 2 mg/kg, and cats with PHA were made using the Kruskal Wallis test followed by paired comparison using the Mann‐Whitney test when relevant. Results are represented graphically by box‐and‐whisker plots. A *P*‐value <.05 was considered significant.

## 3 RESULTS

### 3.1 Healthy cats

The healthy population consisted of 5 spayed females and 5 neutered males (8 domestic short haired, 1 Maine Coon, and 1 Persian) aged 3 years (range, 1‐7). Baseline PAC, potassium concentration, and SBP are presented in Table 1.

**TABLE 1:** Potassium and sodium concentration, systolic blood pressure, aldosterone concentration at T0 and median aldosterone variation rate at T1 and T1.5 in each group of cats.

	Healthy cats receiving placebo	Healthy cats receiving telmisartan 1 mg/kg	Healthy cats receiving telmisartan 2 mg/kg	Cats with PHA	*P*‐value
Baseline potassium concentration	3.9 (3.6; 4.4)	4.3 (3.7; 4.9)	3.9 (3.4; 4.3)	2.5 (2.2; 2.9)	< .001
Baseline sodium concentration	154 (149; 154)	153 (150; 159)	152 (151; 157)	154 (148; 161)	.78
Baseline systolic blood pressure	120 (100; 130)	120 (110; 155)	130 (110; 150)	150 (125; 200)	.05
Baseline aldosterone	283 (65; 1364)	267.7 (105.2; 763.4)	252.6 (167.4; 603.1)	2290 (515; 5488)	.007
Aldosterone variation rate at T1	6% (−39%; +226%)	−24% (−52%; +8%)	−40% (−64%; +5%)	18% (−2%; +49%)	.002
Aldosterone variation rate at T1.5	17% (−29%; 661%)	−22% (−67%; 25%)	−47% (−59%; −15%)	13% (−2%; +34%)	.002
Minimal aldosterone variation rate	5% (−38%; +226%)	−45% (−67%; −5%)	−49% (−64%; −33%)	13% (−2%; +34%)	<.001

*Note*: Data are represented as median (range).

Abbreviation: PHA, primary hyperaldosteronism.

**FIGURE 1:**
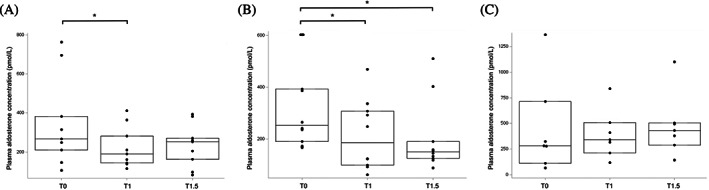
Plasma aldosterone concentration before (T0), 1 hour (T1) and 1.5 hours (T1.5) after oral administration of 1 mg/kg (A, n = 9), 2 mg/kg (B, n = 10) of telmisartan and placebo (C, n = 7) in healthy cats. For each plot, the box represents the interquartile (25th‐75th percentiles) range, the solid horizontal line within the box represents the median. Each filled circle represents the value for an individual cat. Asterisk (*) indicates significant differences.

The PAC was significantly lower at T1 (*P* = .01) after 1 mg/kg telmisartan (Figure 1A), and T1 (*P* = .004) and T1.5 (*P* = .002) after 2 mg/kg telmisartan (Figure 1B), all compared with PAC at T0. No significant change from T0 was identified at T1 and T1.5 after administration of placebo (Figure 1C) or at T1.5 after 1 mg/kg of telmisartan (Figure 1A).

The AVR for 1 mg/kg of telmisartan was −24% (−52%; +9%) at T1 and −22% (−67%; +25%) at T1.5 (Table 1, Figure 3). The AVR for 2 mg/mL telmisartan was −40% (−64%; +5%) at T1 and −47% (−59%; −15%) at T1.5 (Table 1, Figure 3). The AVR associated with the 2 mg/kg telmisartan dose was lower than −33% in all healthy cats and below −40% in 8/10 cats.

After placebo administration, the AVR was +6% (−39%; +226%) at T1 and +17% (−29%; +661%) at T1.5. Among the healthy cats, only 1 cat had an AVR lower than −33% at T1, and none had an AVR lower than −40% at T1 or T1.5 after placebo administration.

No significant changes in plasma potassium concentrations were observed between T0 and T1 or T1.5 after telmisartan (either dosage) or placebo administration. After administration of 1 mg/kg telmisartan, SBP was 120 (110; 155) mm Hg at T0, 120 (100; 130) mm Hg at T1, and 115 (100; 125) mm Hg at T1.5. A significant decrease in SBP was recorded at T1.5 (*P* = .02) as compared to T0. After administration of 2 mg/kg telmisartan, SBP was 130 (110; 150) mm Hg at T0, 115 (60; 140) mm Hg at T1, and 110 (90; 130) mm Hg at T1.5. A significant decrease in SBP was recorded at T1 (*P* = .04) and T1.5 (*P* = .02) as compared with T0. No cat developed clinical signs of systemic hypotension. The lowest SBP observed after telmisartan administration at either dosage was 90 mm Hg and the largest decrease in blood pressure was 40 mm Hg in 1 cat after receiving telmisartan at a dosage of 2 mg/kg. Systolic blood pressure did not change significantly after placebo administration (SBP was 120 [100; 130] mm Hg at T0, 120 [105; 130] mm Hg at T1, and 110 [110; 120] mm Hg at T1.5).

### 3.2 Cats with PHA

The PHA study population consisted of 2 spayed females and 4 neutered males, aged 11 years (11; 18). All cats with PHA were hypokalemic; plasma potassium concentration was 2.5 mmol/L (2.2; 2.9) at study start. The SBP at study start was 150 mm Hg (125; 200) and 2 cats were hypertensive. Ultrasonography detected adrenal masses in 5 cats and unilateral hyperplasia of 1 adrenal gland in 1 cat. Histopathological examination in 2 cats identified adrenal adenocarcinoma. In another cat, cytological examination of a fine needle aspirate of a suspected spleen metastasis was consistent with an adrenal adenocarcinoma. The clinical details of all individuals in the PHA group are presented in Table S1.

Baseline PAC was above the reference interval (14‐258 pmol/L) in all cats with PHA (2290 pmol/L [515; 5488]) and was significantly higher (*P* < .001) than the baseline PAC of healthy cats (274 pmol/L [65; 1364]). In cats with PHA, baseline plasma potassium concentration was significantly lower (*P* < .001) and baseline SBP was significantly higher (*P* = .01) than in healthy cats (Table 1). The PRA was measured in 2 cats and was below the reference interval (60‐630 fmol/L/s) in 1 of them (cat 3, 57 fmol/L/s), and close to the lower end of the reference interval in the other (cat 2, 65 fmol/L/s). The ARR for both these cats was above the reference interval.

**FIGURE 2:**
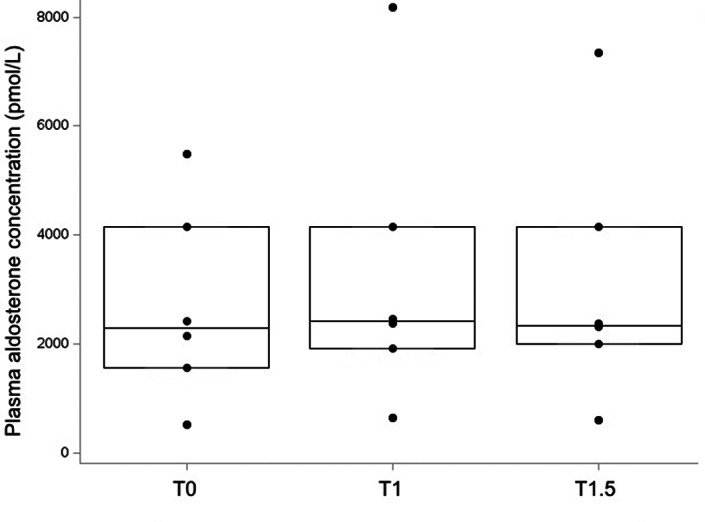
Plasma aldosterone concentration before (T0), 1 hour (T1) and 1.5 hours (T1.5) after oral administration of 2 mg/kg of telmisartan in cats with primary hyperaldosteronism (n = 6, see Figure 1 for legend).

**FIGURE 3:**
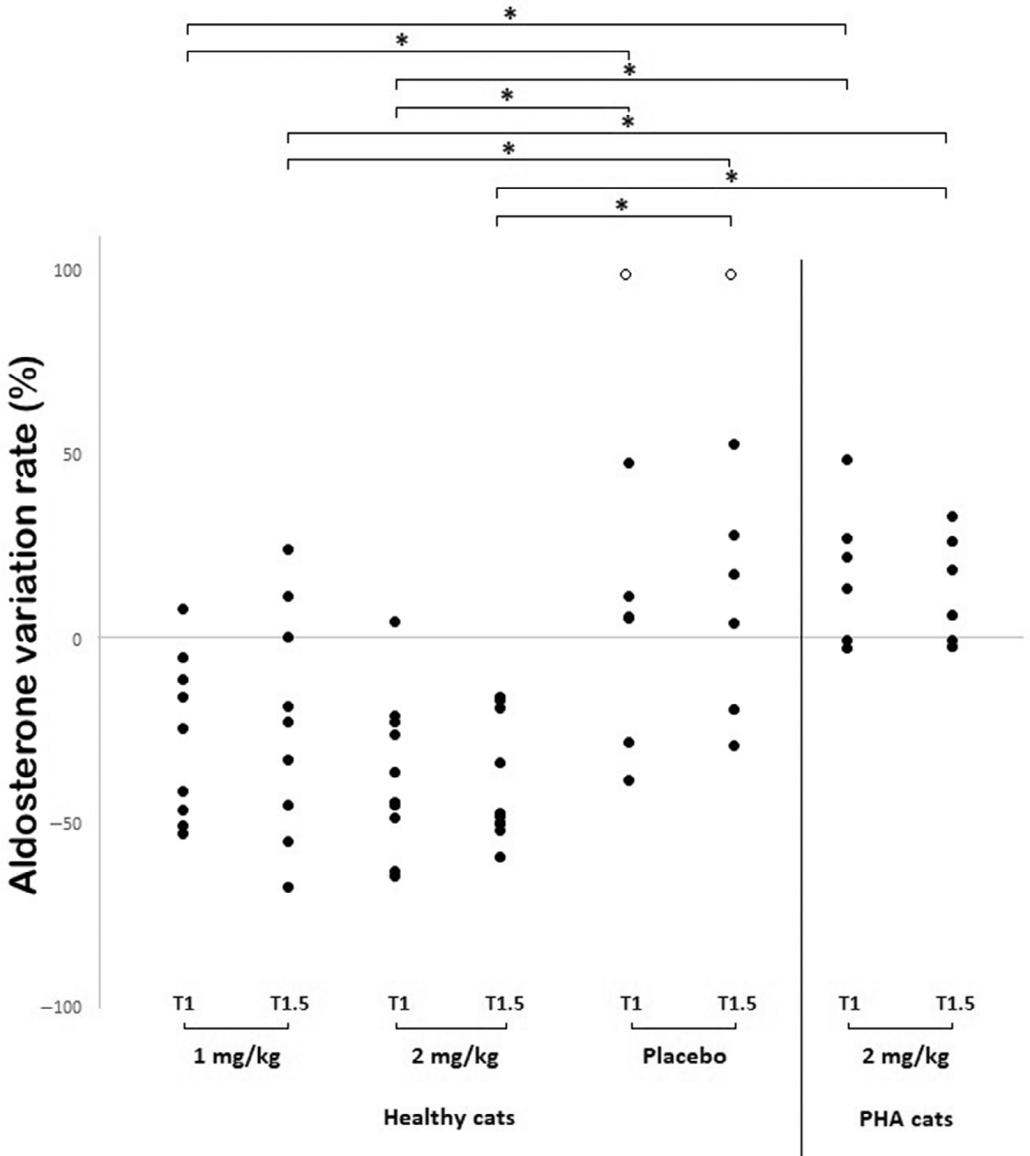
Aldosterone variation rate 1 hour (T1) and 1.5 hours (T1.5) after oral administration of 1 mg/kg or 2 mg/kg of telmisartan or placebo in healthy cats, and of 2 mg/kg of telmisartan in cats with primary hyperaldosteronism (PHA). Each filled circle indicates the value for an individual cat. Unfilled circles are outlier values.

Having obtained the results of telmisartan suppression in healthy cats, the suppression test was performed in cats with PHA using a 2‐mg/kg dose of telmisartan. No significant change in PAC was detected after telmisartan administration in these cats (Figure 2). The AVR was +18% (−2%; +49%) and +13% (−2%; +34%) at T1 and T1.5, respectively. None of the cats with PHA had an AVR lower than −33% (Figure 3, Table 1).

## 4 DISCUSSION

Administration of telmisartan to healthy cats in our study was associated with significant suppression of PAC, whereas no significant effect was associated with placebo administration. A telmisartan dose of 2 mg/kg appeared to be more efficient at decreasing aldosterone concentration (AVR was −40% at T1 and −47% at T1.5) than a 1‐mg/kg dose (AVR was −24% at T1 and −22% at T1.5). The decrease in PAC was also larger with the higher telmisartan dose, although not significantly. We did not identify any absolute value of PAC that could be used to accurately discriminate between cats with and without aldosterone suppression. As far as the relative variation of aldosterone concentration from T0 (ie, AVR) is concerned, it was lower than −33% in all healthy cats at T1 or T1.5 or both when a 2 mg/kg telmisartan dose was chosen. For these reasons, the dosage of 2 mg/kg of telmisartan was chosen to evaluate the telmisartan suppression test in cats with PHA. It could be hypothesized that aldosterone suppression with telmisartan is dose‐dependent, and that a higher telmisartan dosage would be better at discriminating between healthy cats and cats with PHA.

We chose to measure the PAC at T1 and T1.5 because pharmacokinetic studies show that the maximum serum concentration of telmisartan is reached 30 minutes after PO administration (unpublished data). Assuming a plasma half‐life of aldosterone of approximately 15 to 20 minutes (as in humans),^12^ the direct effect of telmisartan on aldosterone concentration is expected to be detectable between 1 and 1.5 hours after PO administration. In our population of healthy cats, aldosterone suppression occurred at T1 or T1.5 or both. Therefore, both sampling times were retained for further evaluation of the telmisartan suppression test in the 6 cats with PHA. It is possible that suppression might be higher at timepoints beyond 1.5 hours after telmisartan dosing, and this possibility needs to be investigated. Because our population of healthy cats was young, we also need to confirm the ability of telmisartan to suppress aldosterone secretion in elderly healthy cats.

Our study confirms the safety of telmisartan when administered to healthy cats. No significant change in plasma potassium concentration was noted in either dose group. Moreover, the anticipated change in potassium concentration secondary to telmisartan administration would constitute hyperkalemia, which should not worsen the condition of cats with PHA. A significant decrease in SBP at T1 occurred after administration of 1 mg/kg of telmisartan, and at T1 and T1.5 after administration of 2 mg/kg of telmisartan, relative to T0. The decreases were not clinically relevant and were consistent with a previous study that demonstrated the tolerability of telmisartan.^10^

A confirmatory test for a diagnosis of PHA must demonstrate that exogenous inhibition of the renin‐angiotensin‐aldosterone system (RAAS) does not suppress aldosterone secretion in cats with PHA. We demonstrated that an oral telmisartan suppression test was rapid, simple, and safe in cats. Telmisartan suppressed PAC in healthy cats, but did not decrease PAC in any of the cats with PHA, even at the higher dosage of 2 mg/kg. In addition, we observed only minimal overlap in AVR between healthy cats and cats with PHA after telmisartan dosing, which suggests that AVR might be another measure of telmisartan response that could confirm a diagnosis of PHA in cats.

Further evaluation of the diagnostic accuracy of the telmisartan suppression test is needed, especially in cats with clinical, biochemical and endocrine changes suggestive of PHA but caused by other diseases stimulating the RAAS. In these cases of secondary hyperaldosteronism, PO telmisartan would be expected to suppress PAC. Specificity of the telmisartan suppression test has thus to be further evaluated. False positive results of suppression tests are known to occur in humans. For example, patients with low‐renin hypertension also show some degree of escape from captotril suppression without suffering from PHA.^13^ Low‐renin hypertension also has been observed in cats,^14^ and it is possible that it could confound the diagnosis of PHA with an oral telmisartan suppression test.

Sensitivity of the telmisartan suppression test in cats also must be evaluated, most notably in clinical situations where underdiagnosis is likely to occur, such as bilateral adrenal hyperplasia. Only 1 cat with PHA presented with adrenal hyperplasia in our study. Given that baseline PAC was only slightly above the reference interval in this cat, diagnosis of PHA may have been missed if PRA had not been measured. Although conclusions cannot be drawn from this single case, it is interesting to note that PO telmisartan did not suppress PAC in this cat and such results provide encouragement for further evaluation of the telmisartan suppression test in this clinical context. Telmisartan‐based suppression tests might however lack sensitivity in some instances. Indeed, many humans with PHA secondary to adrenal adenoma remain responsive to angiotensin II, leading to possible false negative results to sartan‐based suppression tests.^14^ In our study, 3 of 6 cats diagnosed with PHA had adrenal carcinoma, and the histological diagnosis was unknown in the other 3 cats. Additional studies are necessary to evaluate the existence of angiotensin II‐responsive PHA in cats, and eventually to compare the AVR in cats with PHA secondary to adrenal adenoma and adrenal carcinoma.

Our study had some limitations. Firstly, the small sample size could have decreased statistical power to detect differences. Secondly, it would have been interesting to measure PRA in all cats at baseline and at each time point after telmisartan administration because it would have given another indication of the degree of AT1 receptors blockade. Thirdly, because the healthy population included only young cats, extrapolation of the results to a clinical setting, where cats with suspected PHA often are mature adult or elderly cats, must be done with caution. In humans, older age is associated with more autonomous aldosterone secretion and less physiological aldosterone secretion.^15^ Consequently, the absence of aldosterone suppression in the cats with PHA might be age‐related. Additional studies with healthy cats of similar ages to those of cats with PHA therefore are necessary.

## 5 CONCLUSION

We demonstrated that PO administration of telmisartan caused a significant decrease of PAC in healthy young to middle‐aged cats, which was not observed in 6 cats with PHA. Consequently, subject to confirmation of these preliminary results in older healthy cats and in a larger cohort of cats with PHA, the oral telmisartan suppression test may be considered as a simple, safe, and potentially accurate confirmatory test for the diagnosis of PHA in cats.

## CONFLICT OF INTEREST DECLARATION

Authors declare no conflict of interest.

## OFF‐LABEL ANTIMICROBIAL DECLARATION

Authors declare no off‐label use of antimicrobials.

## INSTITUTIONAL ANIMAL CARE AND USE COMMITTEE (IACUC) OR OTHER APPROVAL DECLARATION

Approved by Comité d'éthique en Recherche Clinique (ComERC) Ecole nationale vétérinaire d'Alfort, number 2017‐09‐06.

## HUMAN ETHICS APPROVAL DECLARATION

Authors declare human ethics approval was not needed for this study.

## Supporting information


**Table S1.** Clinical characteristics, main biochemical and diagnostic imaging results, modalities of primary hyperaldosteronism diagnosis in the 6 cats diagnosed with primary hyperaldosteronism. ARR, aldosterone‐to‐renin ratio; NM, neutered male; RR, reference range; SF, spayed female.
