# Supplementary material for: Evaluation of oral telmisartan administration as a suppression test for diagnosis of primary hyperaldosteronism in cats
Source: J Vet Intern Med. 2023 Mar 29;37(4):1341–7. doi: 10.1111/jvim.16689 (PMC10365039; doi:10.1111/jvim.16689)
Supplement: Supplementary file 1 — Table S1. Clinical characteristics, main biochemical and diagnostic imaging results, modalities of primary hyperaldosteronism diagnosis in the 6 cats diagnosed with primary hyperaldosteronism. ARR, aldosterone‐to‐renin ratio; NM, neutered male; RR, reference range; SF, spayed female. [file JVIM-37-1341-s001.pdf]

Table S1 : Clinical characteristics, main biochemical and diagnostic imaging results, modalities of primary hyperaldosteronism diagnosis in the 6 cats diagnosed with primary hyperaldosteronism. NM neutered male, SF spayed female, RR reference range, ARR aldosterone-to-renin ratio.

Case number	Sexe	Age	Clinical signs	Plasma creatinine concentration (mg/L) RR : 8-16 mg/L	Sodium concentration at admission (mmol/L)	Potassium concentration at admission (mmol/L)	Systolic blood pressure at admission (mmHg)	Adrenal ultrasound	Plasma aldosterone concentration (pmol/L), RR : 14-258 pmol/L	Plasma renin activity (fmol/L/s), RR : 60-630 fmol/L/s	ARR, RR : 0-3.8	Histological diagnosis
1	NM	11	Lethargy, inappetence, neck ventroflexion, heart murmur	14.3	161	2.5	150	Right adrenal mass (23 mm)	2 161	ND	ND	Adrenal carcinoma
2	NM	16	Lethargy, weight loss, dehydration, heart murmur	35	155	2.5	125	Left adrenal mass (17 mm)	1 571	65	24.2	ND
3	SF	18	Lethargy, dehydration, paresis, hypertensive retinopathy, heart murmur	8	153	2.9	200	Right adrenal hyperplasia (5.7 mm) and normal left adrenal gland (3.7 mm)	515	57	9.0	ND
4	SF	11	Lethargy, cervical ventroflexion, paresis	8.9	151	2.2	140	Right adrenal mass (60 mm)	> 4155	ND	ND	Adrenal carcinoma
5	NM	11	Lethargy, inappetence, dehydration, heart murmur	36	148	2.2	Not recorded	Left adrenal mass (11 mm)	2419	ND	ND	Adrenal carcinoma
6	NM	11	Decreased appetite, muscle atrophy, heart murmur,	12.2	155	2.6	190	Right adrenal mass (27 mm)	5 488	ND	ND	ND
